# Growth, collapse, and self-organized criticality in complex networks

**DOI:** 10.1038/srep24445

**Published:** 2016-04-15

**Authors:** Yafeng Wang, Huawei Fan, Weijie Lin, Ying-Cheng Lai, Xingang Wang

**Affiliations:** 1 School of Physics and Information Technology, Shaanxi Normal University, Xi’an 710062, China; 2 Department of Physics, Zhejiang University, Hangzhou 310027, China; 3 School of Electrical, Computer, and Energy Engineering, Arizona State University, Tempe, AZ 85287, USA

## Abstract

Network growth is ubiquitous in nature (e.g., biological networks) and technological systems (e.g., modern infrastructures). To understand how certain dynamical behaviors can or cannot persist as the underlying network grows is a problem of increasing importance in complex dynamical systems as well as sustainability science and engineering. We address the question of whether a complex network of nonlinear oscillators can maintain its synchronization stability as it expands. We find that a large scale avalanche over the entire network can be triggered in the sense that the individual nodal dynamics diverges from the synchronous state in a cascading manner within a relatively short time period. In particular, after an initial stage of linear growth, the network typically evolves into a critical state where the addition of a single new node can cause a group of nodes to lose synchronization, leading to synchronization collapse for the entire network. A statistical analysis reveals that the collapse size is approximately algebraically distributed, indicating the emergence of self-organized criticality. We demonstrate the generality of the phenomenon of synchronization collapse using a variety of complex network models, and uncover the underlying dynamical mechanism through an eigenvector analysis.

Growth is a ubiquitous phenomenon in complex systems. Consider, for example, a modern infrastructure in a large metropolitan area. Due to the influx of population, the essential facilities such as the electrical power grids, the roads, water supply, and all kinds of services need to grow accordingly. The issue of how to maintain the performance of the growing systems under certain constraints (e.g., quality of living) becomes critically important from the standpoint of sustainability. To develop a comprehensive theoretical framework to understand, at a quantitative level, the fundamental dynamics of sustainability in complex systems subject to continuous growth is a challenging and open problem at the present. In this paper, to shed light on how a complex network can maintain its function and how such a function may be lost during network growth, we focus on the dynamics of synchronization. In particular, if a small network is synchronizable, as it grows in size the synchronous state may collapse. The main purpose of the paper is to uncover and understand the dynamical features of synchronization collapse as the network grows. As will be explained, our main result is that the collapse is essentially a self-organizing dynamical process towards criticality with an algebraic scaling behavior.

From the beginning of modern network science, growth has been recognized and treated as an intrinsic property of complex networks^1^,^2^. For example, the pioneering model of scale free networks^3^ had growth as a fundamental ingredient to generate the algebraic degree distribution. The growth aspect of this model is, however, somewhat simplistic as it stipulates a monotonic increasing behavior in the network size, whereas the growth behavior in real world networks can be highly non-monotonic. For example, in technological networks such as the electric power grid, introducing a new node (e.g., a power station) will increase the load on the existing nodes in the network, which can trigger a cascade of failures when overload occurs^4^,^5^,^6^,^7^,^8^,^9^,^10^,^11^,^12^,^13^,^14^,^15^,^16^,^17^,^18^,^19^,^20^,^21^,^22^,^23^,^24^. In this case, the addition of a new node does not increase the network size but instead results in a network collapse^5^,^24^. A similar phenomenon was also observed in ecological networks, where the introduction of a new species may result in the extinction of many existing species^25^,^26^. In an economic crisis, the failure of one financial institute can result in failures of many others in a cascading manner^21^,^27^. To take into account the phenomenon of non-monotonic network growth so as to avoid network collapse, an earlier approach was to constrain the growth according to certain functional requirement such as the system stability with respect to certain performance, i.e., to impose the criterion that the system must be stable at all times^25^. It was revealed that network growth subject to a global stability constraint can lead to a non-monotonic network growth without collapse^28^. Constraint based on network synchronization was proposed^29^, where it was demonstrated that imposing synchronization stability can result in a highly selective and dynamic growth process^29^ in the sense that it often takes many time steps for a new node to be successfully “absorbed” into the existing network.

To be concrete, we study the growth of complex networks under the constraint of synchronization stability. Synchronization of coupled nonlinear oscillators has been an active area of research in nonlinear science^30^,^31^,^32^,^33^,^34^, and it is an important type of collective dynamics on complex networks^35^. Earlier studies focused on systems of regular coupling structures, e.g., lattices or globally coupled networks. The discovery of the small world^36^ and scale free^3^ network topologies in realistic systems generated a great deal of interest in studying the interplay between complex network structure and synchronization^37^,^38^,^39^,^40^,^41^,^42^,^43^,^44^,^45^,^46^,^47^,^48^,^49^,^50^,^51^. Since the structures of many realistic networks are not static but evolving with time^52^,^53^, synchronization in time-varying complex networks was also studied^54^,^55^,^56^ to reveal the dynamical interplay between the time-dependent network structure and synchronization^39^,^57^,^58^. We note that there was a line of works addressing the improvement of synchronization by evolving the network structure, such as link rewiring^59^,^60^, adjustment of coupling weights^61^,^62^, change the coupling scheme^63^,^64^, but in these works the network size is assumed to be fixed.

To investigate the growth of stability-constrained complex networks, a key issue is the different time scales involved in the dynamical evolution^28^,^29^,^65^. For network growth constrained by synchronization, in general there are two key time scales: one associated with the transient synchronization dynamics occurred in a static network, denoted as *T*_*s*_, and another characterizing the speed of network growth, e.g., the time interval between two successive nodal additions, *T*_*g*_. The interplay between the two time scales can result in distinct network evolution dynamics. For example, for *T*_*s*_ ≫ *T*_*g*_, the stability constraint would have little effect on the network evolution and, in an approximate sense, the network grows as if no constraint were imposed. However, for *T*_*s*_ ≪ *T*_*g*_, the network remains synchronized at all times. In particular, since the stability is determined by the network structure, e.g., through the eigenvalues of the coupling matrix, the dynamics of network evolution is effectively decoupled from that of synchronization.

For *T*_*s*_ ≈ *T*_*g*_, complicated network evolution dynamics can arise^65^, where the two types of dynamical processes, i.e., growth and synchronization, are entangled. Depending on the instant network structure and synchronization behavior, the addition of a new node may either increase or decrease the network size. For example, if the new node induces a desynchronization avalanche, a number of nodes will be removed if their synchronization errors exceed some threshold values, resulting in a sudden decrease of the network size and potentially a large scale collapse. In this paper, we focus on the regime of *T*_*s*_ ≈ *T*_*g*_ and introduce a tolerance threshold to determine if a node has become desynchronized. Specifically, after each transient period of evolution, we remove all nodes with synchronization error exceeding this threshold. During the course of evolution, the network can collapse at random times. Strikingly, we find that the size of the collapses follows an algebraic scaling law, indicating that the network growth dynamics under the synchronization constraint can be regarded as a process towards self-organized criticality (SOC).

## Results

### Model of network growth subject to synchronization constraint

We consider the standard scale-free growth model^3^ but impose a synchronization-based constraint for nodal removal. Specifically, starting from a small, synchronizable core of *m*_0_ coupled nonlinear oscillators (nodes), at each time step *n*_*g*_ of network growth, we add a new node with random initial condition into the network. The new node is connected to *m* existing nodes according to the preferential attachment probability Π_*i*_ = *k*_*i*_/∑_*j*_*k*_*j*_, where *i*, *j* = 1, 2, …, *n* are the nodal indices and *k*_*i*_ is the degree of the *i*th node. We then monitor the system evolution for a fixed time period (*T*_*g*_) and calculate the nodal synchronization error *δr*_*i*_ (to be defined below). Defining *δr*_*c*_ as the tolerance threshold for nodal desynchronization, if all nodes in the network meet the condition *δr*_*i*_ < *δr*_*c*_, the network size will be increased by one. Otherwise, the nodes with *δr*_*i*_ > *δr*_*c*_ will be removed from the network, together with the links attached to them. For convenience, we use the term “collapse” to describe the process of nodal removal and the number of removed nodes, Δ*n*, is the collapse size.

For simplicity, we set the nodal dynamics to be identical and adopt the normalized coupling scheme^66^,^67^, where the dynamical evolution of the *i*th oscillator in the network is governed by


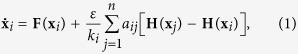


with **F** and **H** representing, respectively, the dynamics of the isolated oscillator and the coupling function. The network structure is characterized by the adjacency matrix {*a*_*ij*_}, where *a*_*ij*_ = 1 if oscillators *i* and *j* are directly connected, and *a*_*ij*_ = 0 otherwise. The parameter *ε* > 0 is the uniform coupling strength. Note that the coupling strength from node *j* to node *i*, *c*_*ij*_ = (*εa*_*ij*_)/*k*_*i*_, in general is different from that for the opposite direction, so the network is weighted and directed^67^. The class of models of linearly coupled nonlinear oscillators with variants are commonly used in the literature of network synchronization^68^. While Eq. (1) is for continuous-time dynamical systems, networks of coupled nonlinear maps can be formulated in a similar way.

To be concrete, we assume that the individual nodal dynamical process is described by the chaotic logistic map, *x*(*t* + 1) = *F*[*x*(*t*)] = 4*x*(*t*)[1 − *x*(*t*)], and choose *H*(*x*) = *F*(*x*) as the coupling function. The coupling strength is fixed at *ε* = 1. The initial network consists of *m*_0_ = 8 globally coupled nodes, which is synchronizable for the given coupling strength. For a fixed time interval *T*_*g*_ = 300, we introduce a new node (map) into the network with a randomly chosen initial condition in the interval (0, 1) by attaching it to the existing nodes according to the preferential attachment rule. The synchronization error is defined as *δr*_*i*_ = |*x*_*i*_ − 〈*x*〉| ith 〈*x*〉 = ∑_*i*_*x*_*i*_/*n* being the network-averaged state, which is calculated at the end of each time interval *T*_*g*_. We set the tolerance threshold to be *δr*_*c*_ = 10^−10^ (somewhat arbitrarily). The growing process is terminated either if the network has completely collapsed (*n* ≈ 0) or when its size reaches a preset upper bound (e.g, 1000).

**Figure 1:**
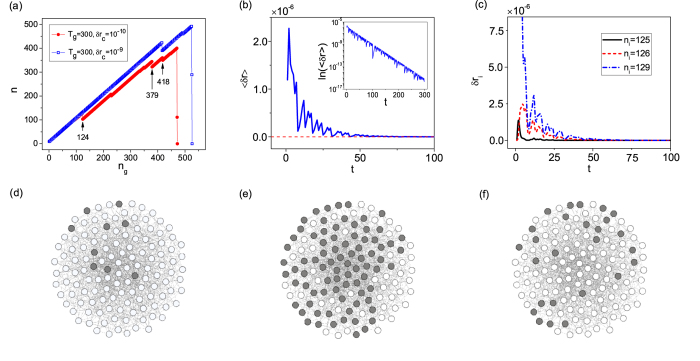
Evolution of a network of coupled chaotic logistic maps subject to synchronization constraint. The transient period for network to be synchronized is *T*_*g*_ = 300, and the tolerance threshold for desynchronization at the nodal level is *δr*_*c*_ = 10^−10^. (**a**) Variation of the network size, *n*, with the time step of node addition, *n*_*g*_. The (red) filled circles are the results for *T*_*g*_ = 300 and *δr*_*c*_ = 10^−10^, and the (blue) open squares are for *T*_*g*_ = 300 and *δr*_*c*_ = 10^−9^. (**b**) Time evolution of the network averaged synchronization error, 〈*δr*〉. Inset: the corresponding semi-logarithmic plot. (**c**) Time evolution of the synchronization error, *δr*_*i*_, for three typical nodes in the network. (**d**–**f**) Snapshots of the nodal synchronization errors, *δr*_*i*_, for three different time instants: (**d**) *t* = 123*T*_*g*_ + 1, (**e**) *t* = 123*T*_*g*_ + 5, and (**f**) *t* = 124*T*_*g*_. Nodes with *δr* > *δr*_*c*_ are represented by filled circles.

Figure 1 shows the network size *n* versus the time step *n*_*g*_. We see that, after an initial period of linear growth (*n*_*g*_ ≤ 123), the network size is suddenly decreased from *n* = 128 to 103, signifying that a collapse event of size Δ*n* = 25 has occurred after the addition of the 124^th^ oscillator. After the collapse, the network begins to expand again. In the subsequent time evolution, collapse of different sizes occurs at random times, e.g., Δ*n* = 22 at *n*_*g*_ = 379 and Δ*n* = 10 at *n*_*g*_ = 418. For relatively small network size, when a collapse event occurs, the removed nodes account for only a small fraction of the nodes in the entire network (e.g., Δ*n*/*n* < 10%), with growth followed immediately after the collapse. However, as the network size exceeds a critical value, say *n*_*max*_ = 400, this scenario of small-scale collapse followed by growth is changed dramatically. As shown in Fig. 1(a), for *n*_*g*_ = 471, a catastrophic collapse event occurs, which removes over 75% of the nodes in the network (from 471 to 111). More strikingly, there is no growth after the event - the network continues to collapse. At the end of *n*_*g*_ = 472, not a single node remains in the network, i.e., the network has collapsed *completely*.

To gain more insights into the dynamics of network collapse, we monitor the system evolution for the time period 123*T*_*g*_ < *t* < 124*T*_*g*_, i.e., the response of the network dynamics to the addition of the 124^th^ node. Figure 1(b) shows the time evolution of the averaged network synchronization error, 〈*δr*〉 = ∑_*i*_*δr*_*i*_/*n*, where its value approaches zero rapid with time. A semi-logarithmic plot reveals an exponentially decreasing behavior for 〈*δr*〉 [inset of Fig. 1(b)], indicating that the network is able to restore synchronization for relatively large values of *T*_*g*_. However, for *T*_*g*_ = 300, at the end of the time interval *t* = 124*T*_*g*_, the synchronization errors of certain nodes exceed the threshold, leading to their removal from the network. The synchronization errors for three typical nodes are shown in Fig. 1(c). Examining the individual nodal synchronization errors *δr*_*i*_, we find that, the “disturbance” triggered by the addition of a new node spreads quickly over the network, as shown in Fig. 1(d). After the disturbance reaches the maximal dynamical range at *t* ≈ 123*T*_*g*_ + 5 [Fig. 1(e)], it begins to shrink and, at the end of this time interval, there are still a few nodes with *δr* > *δr*_*c*_, as shown in Fig. 1(f). Based on their dynamical responses, the nodes can be roughly divided into three categories, as shown in Fig. 1(c). Specifically, for most nodes, as time increases *δr* first increases and then decreases, e.g., the 126^th^ node. There are also nodes for which the values of *δr* decrease monotonically with time, e.g., the 125^th^ node. Finally, there are a few nodes for which the values of *δr* remain about 0, e.g., the 129^th^ node. We also observe that, sometimes, the new node, whose introduction into the network triggers a network collapse, in fact remains in the network.

### Statistical properties of collapse and self-organized criticality

In terms of practical significance, the following questions about network collapse are of interest: (1) what kind of nodes are more likely to be removed? (2) what is the size distribution of the collapse? (3) how frequent is the network collapsed? and (4) what are the effects of the tolerance threshold *δr*_*c*_ and growing interval *T*_*g*_ on the collapse? In what follows, we address these questions numerically.

**Figure 2:**
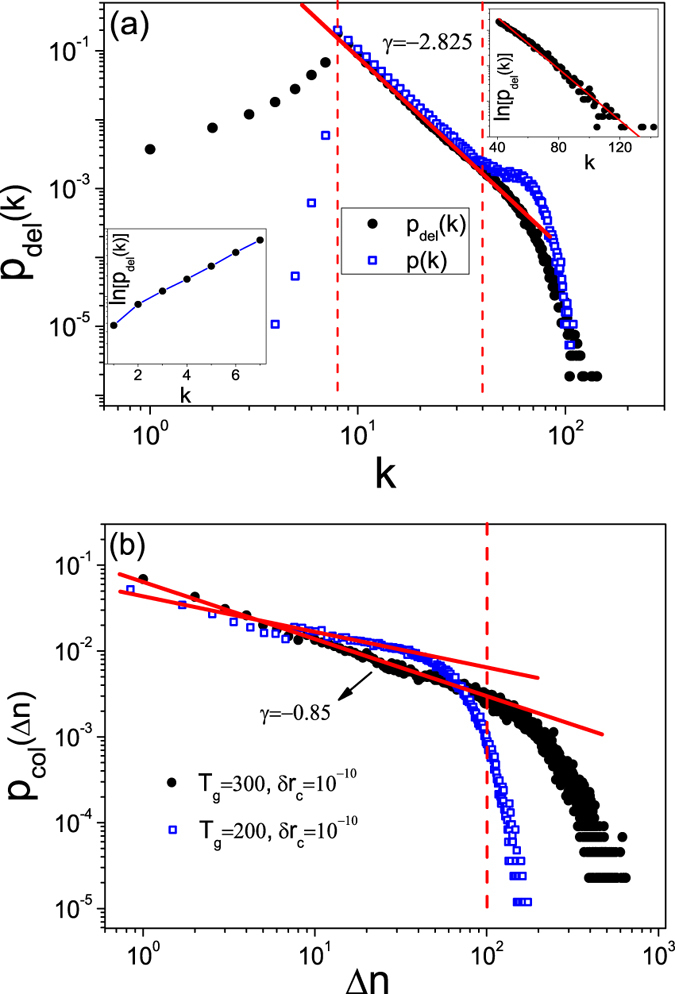
Statistical properties of collapse and SOC. (**a**) Degree distribution *p*_*del*_(*k*) of the removed nodes (filled circles). For *k* ∈ [*m*, 40], the scaling behavior is *p*_*del*_(*k*) ~ *k*^*γ*^, with *γ* ≈ −2.83. For *k* ≤ *m* and *k* ≥ 40, *p*_*del*_(*k*) increases and decreases with *k* exponentially, respectively. Open squares are for the degree distribution *p*(*k*) of the generated network. (**b**) Size distribution *p*_*col*_(Δ*n*) of the collapse event for parameters *T*_*g*_ = 300 and *δr*_*c*_ = 10^−10^. For Δ*n* ∈ [1,100], the scaling is *p*_*col*_(Δ*n*) ~ Δ*n*^*γ*^ with *γ* ≈ −0.85. Open squares are for the size distribution of the collapse events for *T*_*g*_ = 200 and *δr*_*c*_ = 10^−10^. The algebraic scaling of the collapse size signifies SOC. The results are averaged over 100 network realizations.

A simple way to identify the removed nodes is to examine their degrees. With the same parameters as in Fig. 1, we plot in Fig. 2(a) the normalized degree distribution, *p*_*del*_(*k*), of the removed nodes collected from a large number of collapse events (except the catastrophic one that totally destroys the network). We see that the distribution contains approximately three distinct segments with different scaling behaviors. Specifically, for *k* ∈ [1, *m*], *p*_*del*_(*k*) increases with *k* exponentially. For *k* ∈ [*m*, 40], *p*_*del*_(*k*) decreases with *k* algebraically with the exponent *γ* ≈ −2.83. For *k* ∈ [40, 120], *p*_*del*_(*k*) decreases with *k* exponentially. Since, in our model each new node has *m* = 8 links, it is somewhat surprising to see from Fig. 2(a) that some nodes have their degrees smaller than *m*. This phenomenon can be attributed to the node removal mechanism: when a node is removed, all links associated to it are also removed. Another phenomenon is that *p*_*del*_(*k*) reaches its maximum at *k* = 8, which seems to contradict the previous result that nodes of large degrees are more stable with respect to synchronization than those of small degrees^61^,^66^,^67^,^68^.

Since *p*_*del*_(*k*) is obtained from a large number of collapses, to uncover the interplay between nodal stability and degree, we need to take into account the degree distribution *p*(*k*) of the generated network. To find *p*(*k*), we use the largest network emerged in the growth process (the network formed immediately before the catastrophic collapse) and obtain the degree distribution for an ensemble of such networks. The results are also shown in Fig. 2(a). We see that the two distributions, *p*_*del*_(*k*) and *p*(*k*), coincide with each other well, where *p*(*k*) also contains three distinct segments and reaches its maximum at *k* = *m*. The consistency between *p*_*del*_(*k*) and *p*(*k*) suggests that the nodal stability is independent of the degree. Statistically, we thus expect that the small and large degree nodes to have equal probability to be removed.

Figure 2(b) shows the collapse size distribution, where the catastrophic network size *n*_*max*_ is not included. We see that, in the interval Δ*n* ∈ [1, 100], the distribution follows an algebraic scaling: *p*_*col*_(Δ*n*) ~ Δ*n*^*γ*^, with *γ* ≈ −0.85. For Δ*n* > 100, an exponential tail is observed. To test whether the exponential tail is a result of the finite size effect, we decrease the transient period to *T*_*g*_ = 200 and plot the distribution of the collapse size again. (As we will demonstrate later, as *T*_*g*_ is decreased, the maximum network size *n*_*max*_ will decrease monotonically.) Figure 2(b) indicates that, comparing with the case of *T*_*g*_ = 300, the regime of algebraic scaling is shifted toward the left for *T*_*g*_ = 200. Specifically, for *T*_*g*_ = 200, we have *p*_*col*_(Δ*n*) ~ Δ*n*^*γ*^ in the interval Δ*n* ∈ [1,50], where the fitted exponent is about −0.79.

The emergence of algebraic scaling in the size distribution of network collapse is interesting from the viewpoint of SOC that occurs in many real-world complex systems. For a dynamical system subject to continuous external perturbations, during its evolution towards SOC, it can appear stable for a long period of time before a catastrophic event occurs, and the probability for the catastrophe can be markedly larger than intuitively expected (algebraic versus exponential scaling)^69^,^70^. In our case, there is a long time period of synchronization stability in spite of the small-size collapses, but catastrophic collapses that remove all or most of the nodes in the network can occur, albeit rarely. There are a variety of models for SOC, but the unique feature of our model is that it exploits network synchronization stability as a mechanism for catastrophic failures. Since synchronization is ubiquitous in natural and man-made complex systems, the finding of SOC in synchronization-stability-constrained network may have broad implications. For instance, synchronization is commonly regarded as the dynamical basis for normal functioning of the power grids^71^, and there is empirical evidence that the size of the blackouts follows roughly an algebraic distribution^72^.

**Figure 3:**
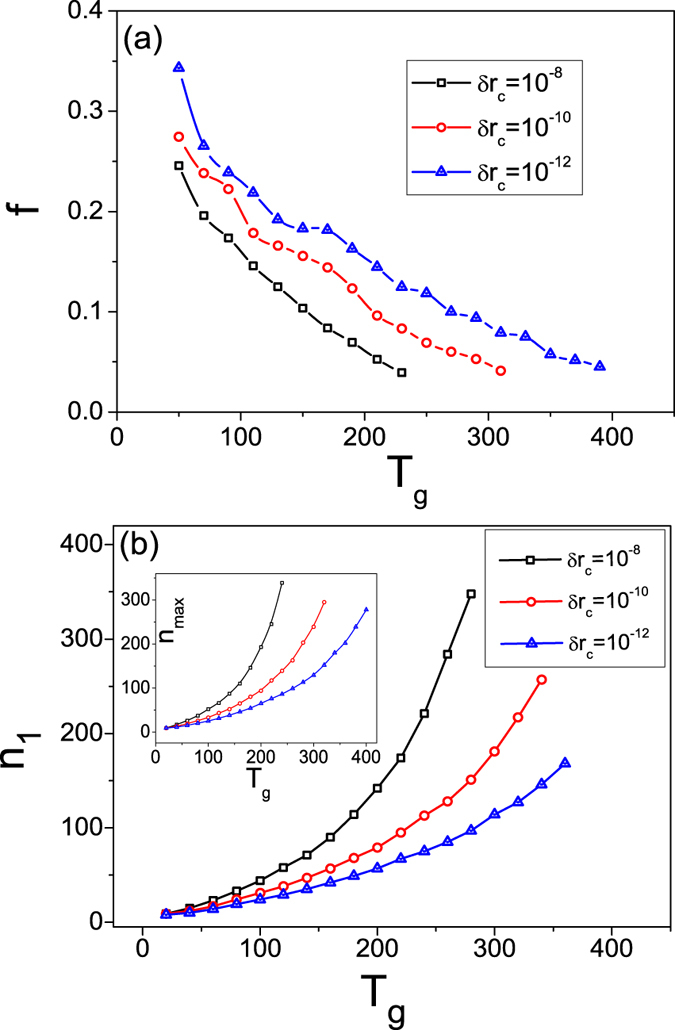
Behavior of the collapse frequency. (**a**) The collapse frequency *f* as a function of the transient interval *T*_*g*_ for different values of the tolerance threshold *δr*_*c*_. (**b**) The first critical network size *n*_1_ versus *T*_*g*_ for different values of *δr*_*c*_. Inset: dependence of the maximum network size *n*_*max*_ on *T*_*g*_. The results are averaged over 100 network realizations.

We proceed to study the frequency of network collapse. Let Δ*n*′ be the period of continuous network growth, i.e., the number of nodes successively added into the network between two adjacent collapses. The collapse frequency is *f* = 1/〈Δ*n*′〉, where 〈Δ*n*′〉 is the averaged period. For the same parameters in Fig. 1, we find *f* ≈ 1/21. That is, on average the network collapses every 21 new additions. Since the synchronization errors are evaluated at the end of each transient interval and nodes are removed according to a predefined tolerance threshold, we expect the collapse frequency to depend on the parameters *T*_*g*_ and *δr*_*c*_. This is apparent in Fig. 1(a), where the network growth under the parameters *T*_*g*_ = 300 and *δr*_*c*_ = 10^−9^ is also shown. We see that, comparing with the case of *δr*_*c*_ = 10^−10^, the catastrophic collapse is postponed. To assess the influence of *T*_*g*_ and *δr*_*c*_ on *f*, we show in Fig. 3(a) *f* versus *T*_*g*_ for different values of *δr*_*c*_. It can be seen that, with the increase of *T*_*g*_ or *δr*_*c*_, *f* decreases monotonically.

For the process of network growth, two particularly relevant quantities are: (1) the critical network size *n*_1_ at which the first collapse occurs and (2) the maximum network size *n*_*max*_ beyond which a catastrophic collapse occurs. Similar to the collapse frequency, these two quantities depend on the parameters *T*_*g*_ and *δr*_*c*_. Figure 3(b) shows *n*_1_ (*n*_*max*_) versus *T*_*g*_ for different values of *δr*_*c*_. We see that, as *T*_*g*_ or *δr*_*c*_ is increased, *n*_1_ (*n*_*max*_) increases monotonically. That is, by increasing *T*_*g*_ or *δr*_*c*_, one can postpone the first and the catastrophic network collapse but eventually it will occur.

### Physical theory of synchronization based network collapse

**Figure 4:**
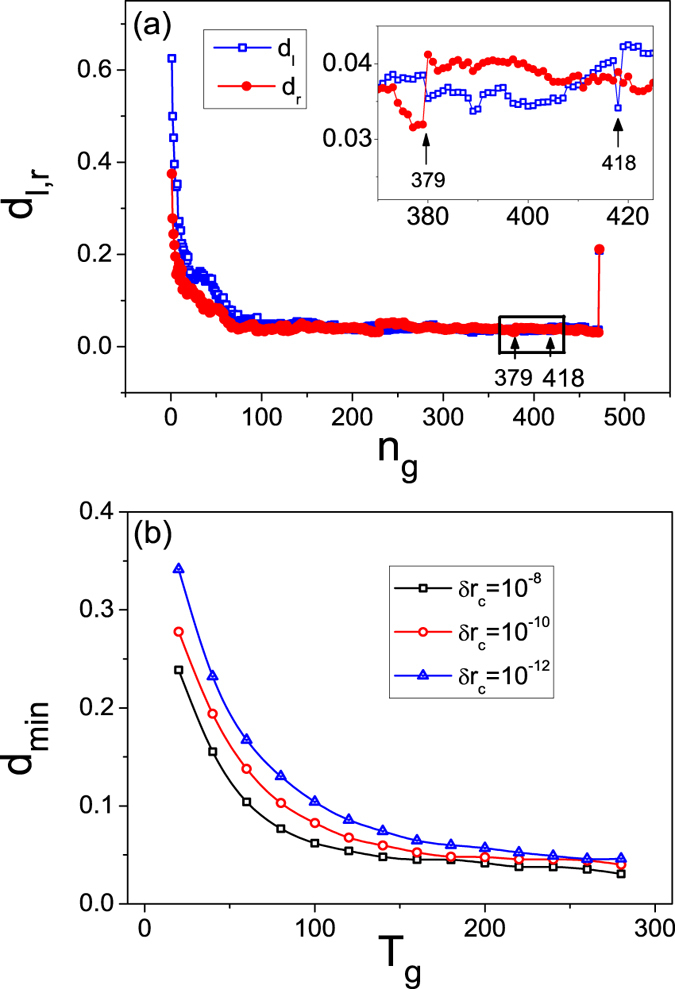
Behavior of synchronization distances. (**a**) Time evolution of the stability distances *d*_*l*,*r*_. Inset: a magnification of part of the evolution. (**b**) The smallest stability distance *d*_*min*_ versus the transient interval *T*_*g*_ for different values of the tolerance threshold *δr*_*c*_. The results are averaged over 100 network realizations.

As the network synchronizability can be characterized by the stability distances *d*_*l*,*r*_ (see Methods), we calculate the evolution of *d*_*l*,*r*_ during the course of network growth, as shown in Fig. 4(a). In accordance with the process of network growth [Fig. 1], the time evolution of *d*_*l*,*r*_ also consists of distinct regimes. Firstly, as *n*_*g*_ increase from 1 to 123, *d*_*l*,*r*_ approaches zero quickly. Secondly, in the interval *n*_*g*_ ∈ (123, 470), *d*_*l*,*r*_ remains about zero. A magnification of this interval reveals that, while *d*_*l*,*r*_ tend to reach zero, the process is occasionally interrupted by some small increments. Checking the points at which *d*_*l*,*r*_ increase suddenly [inset of Fig. 4(a)], we find that these points correspond to exactly the time instants of network collapses. For example, for *n*_*g*_ = 379, *d*_*l*_ increases from 0.032 to 0.041 [Fig. 4(a)], while at the same time there is a collapse event in which the network size changes from *n* = 344 to 322 [Fig. 1(a)]. Finally, at the critical instant *n*_*g*_ = 472 where the catastrophic collapse occurs, *d*_*l*_ and *d*_*r*_ change suddenly to 0.21 and 0.22, respectively.

Figure 4 thus indicates that, for the entire process of network growth, the stability distances *d*_*l*,*r*_ remain positive so that the network is synchronizable at all time. That is, even at the time when a collapse occurs, no node would be removed if the transient time *T*_*g*_ is sufficiently long. It may then be said that, with respect to the impact of the network synchronizability (as determined by the network structure), network collapse is equally influenced by the transient synchronization dynamics. Increasing *T*_*g*_ can thus effectively postpone the collapses as the network grows, a manifestation of which is a further decrease in *d*_*l*,*r*_ at the collapses. Let *d*_*min*_ be the minimum of *d*_*l*,*r*_ during the process of network growth. Figure 4(b) shows *d*_*min*_ versus *T*_*g*_ for different values of *δr*_*c*_. As anticipated, increasing the value of *T*_*g*_ or *δr*_*c*_ results in a monotonic decrease in the value of *d*_*min*_, which agrees with the results of direct simulations in Fig. 3(b) where a postponement of the catastrophic collapse is explicitly demonstrated.

**Figure 5:**
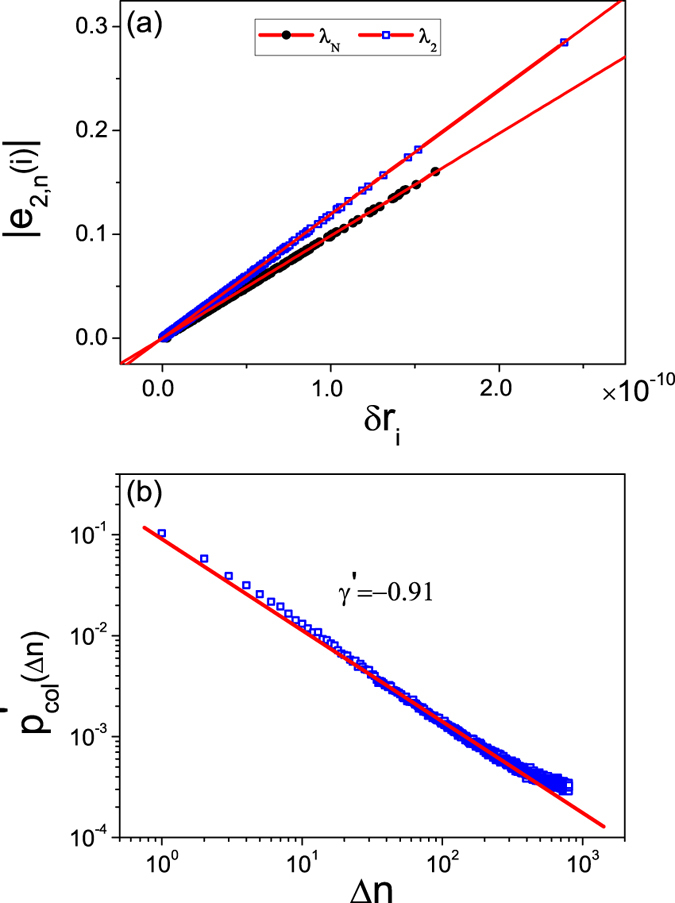
Relation between key eigenvector and synchronization error. (**a**) The linear relationship between the absolute eigenvector elements |*e*_2,*n*_(*i*)| and the oscillator synchronization errors *δr*_*i*_ at different steps of the network growth. Filled circles are for the case of *n*_*g*_ = 418, Λ(*σ*_2_) > Λ(*σ*_*n*_), where the relation |*e*_2_(*i*)| ~ *δr*_*i*_ holds. Open squares specify the case of *n*_*g*_ = 379 and Λ(*σ*_2_) < Λ(*σ*_*n*_) where we have |*e*_*n*_(*i*)| ~ *δr*_*i*_. (**b**) Size distribution of network collapse predicted from the eigenvector analysis. The distribution follows an algebraic scaling law: 
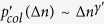
, with the fitted exponent being *γ*′ ≈ −0.91.

The fact that *d*_*l*,*r*_ become approximately zero prior to a catastrophic collapse implies that the network becomes marginally stable during the growing process, i.e., the oscillator trajectories deviate only slightly from the synchronous manifold. In this case, desynchronization is determined by the two extreme modes, *σ*_2_ and *σ*_*n*_, as the corresponding transverse Lyapunov exponents Λ(*σ*_2,*n*_) are larger than those associated with other transverse modes^73^. This feature makes possible a theoretical analysis of the collapse phenomenon. In particular, assuming *d*_*l*,*r*_ ≈ 0 and Λ(*σ*_2_) > Λ(*σ*_*n*_) (so that the 2^nd^ transverse mode is more unstable), we have that desynchronization is mainly determined by the 2^nd^ mode, with *ξ*_2_(*t*) ~ exp[Λ(*σ*_2_)*t*]. Since Λ(*σ*_2_) ≈ 0, we have *ξ*_2_(*t*) ~ Λ(*σ*_2_)*t* . Transforming this mode back to the nodal space, we obtain *δr*_*i*_ = |*e*_2,*i*_*ξ*_2|_ ~ |*e*_2,*i*_Λ(*σ*_2_)*t*, where *e*_2,*i*_ is the *i*th component of the eigenvector ***e***_2_ associated with *σ*_2_. For the given network structure, the value of Λ(*σ*_2_) is fixed. We thus have


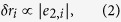


which establishes a connection between the network structure and the oscillator stability. It is only necessary to calculate the eigenvector associated with the most unstable mode to identify the unstable oscillators,

Relation (2) can be verified numerically. As shown in the inset of Fig. 4(a), at the growing step *n*_*g*_ = 379, the network contains *n* = 322 oscillators and the two extreme eigenvalues are (*σ*_2_,*σ*_*n*_) = (0.538, 1.468). Since Λ(*σ*_2_) = −0.079 and Λ(*σ*_*n*_) = −0.066, desynchronization is determined by the *n*th mode. Figure 5(a) shows the synchronization errors (measured at the end of the 379^th^ growing step) *δr*_*i*_ versus the absolute eigenvector element |*e*_2,*i*_| for all the oscillators in the network, which is obtained from the network coupling matrix *C*. We see that *δr*_*i*_ increases with |*e*_*n*,*i*_| linearly. The linear relationship is also observed when the 2^nd^ transverse mode is more unstable. For example, at the growing step *n*_*g*_ = 418, the network contains *n* = 350 oscillators and the two pertinent Lyapunov exponents are [Λ(*σ*_2_), Λ(*σ*_*n*_)] = (−0.070, −0.081). The linear variation of *δr*_*i*_ with |*e*_2,*i*_| is also shown in Fig. 5(a).

Relation (2) can also be used to interpret the size distribution of the network collapses observed numerically [e.g., Fig. 2(b)]. Let *δr*_*i*_(0) be the initial synchronization error of the *i*th oscillator induced by the newly added oscillator. After a transient phase of length *T*_*g*_, the error becomes *δr*_*i*_ ≈ *δr*_*i*_(0)|*e*_*j*′,*i*_|*exp*[Λ(*σ*_*j*′_)*T*_*g*_], with *j*′ = 2 or *n* (depending on which mode is more unstable). As Λ(*σ*_*j*′_) is approximately zero, we have *δr*_*i*_ ≈ *δr*_*i*_(0)|*e*_*j*′,*i*_|[1 + Λ(*σ*_*j*′_)*T*_*g*_]. Setting *δr*_*i*_ = *δr*_*c*_, we get the critical element


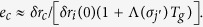


Thus, whether the *i*th oscillator is removed solely depends on the element *e*_*j*′,*i*_. In particular, if |*e*_*j*′,*i*_| > *e*_*c*_, we have *δr*_*i*_ > *δr*_*c*_ so that the oscillator will be removed; otherwise it will remain in the network. Assuming the oscillators have the same initial error *δr*(0), we can estimate the size of the network collapse simply by counting the number of elements satisfying the inequality |*e*_*j*′,*i*_| > *e*_*c*_. To verify this idea, we generate scale-free networks, calculate the eigenvector ***e***_2_, and identify the largest element *e*_*max*_ of ***e***_2_. Choosing *e*_*c*_ randomly from the range (0, *e*_*max*_) [since *d*(0) is dependent upon the (random) initial condition of the newly added oscillator], we truncate the eigenvector elements, where the number of truncated elements is the collapse size. We repeat this truncation procedure for a large number of statistical realizations and calculate the size distribution of the collapses. The result for a network of size *n* = 800 is shown in Fig. 5(b). We see that the size distribution calculated from the eigenvector analysis also follows an algebraic scaling: 
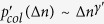
, where the fitted exponent is *γ*′ ≈ −0.91. This is in good agreement with the one obtained from direct simulations [Fig. 2(b)], where the algebraic scaling exponent is *γ* ≈ −0.85 for the interval Δ*n* ∈ [1, 100].

### Alternative models of network dynamics

To demonstrate the generality of the synchronization based network collapse phenomenon and its SOC characteristics, we simulate continuous time dynamics on networks that grow according to alternative rules other than the preferential attachment mechanism. In fact, in network modeling, the way by which a new node is added to the existing network can have a determining role in the network structure^1^. For example, in unconstrained growing networks, random attachment cannot lead to any scale free feature but results in an exponential degree distribution^74^. Since the network structure has a significant effect on synchronization, we expect the characteristics of network growth dynamics following random attachment to be different from those from the preferential attachment rule. Besides the network structure, our eigenvector analysis indicates that the synchronization behavior is also dependent upon the nodal dynamics and the coupling function. For example, for a different type of nodal dynamics, the MSF curve can be dramatically different, so is the stability parameter region^75^,^76^,^77^. We are led by these considerations to study continuous-time oscillator networks that grow according to the random attachment rule. For the coupled logistic map network with the random attachment rule, the dynamical and statistical properties of network growth are found to be similar to those with the preferential attachment mechanism. In particular, the size of the collapse event follows approximately an algebraic scaling, where the scaling exponent decreases with the value of the synchronization threshold.

**Figure 6:**
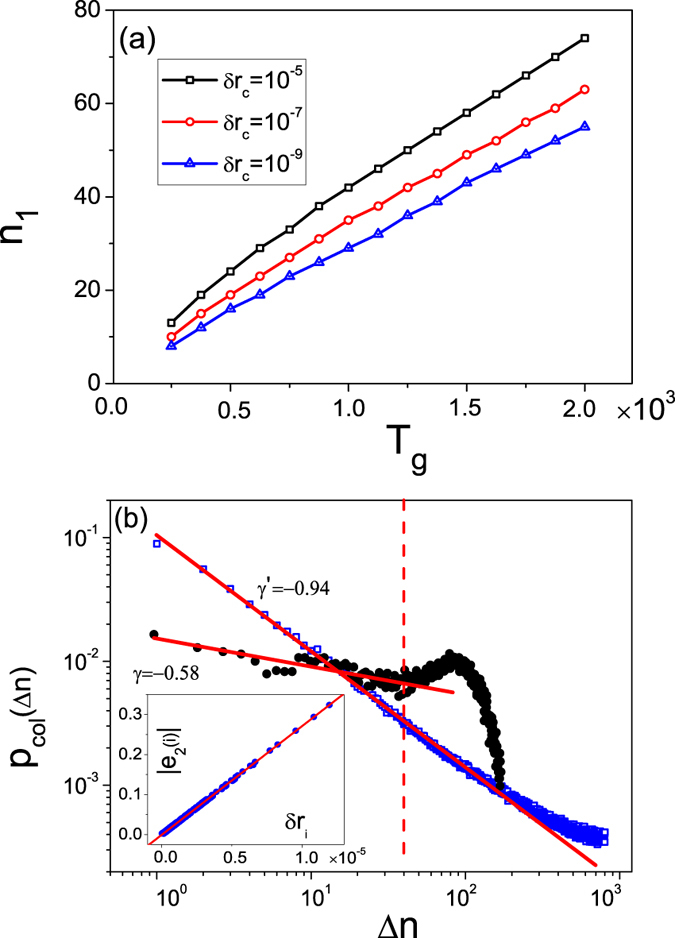
Synchronization based collapse in networks of continuous-time nonlinear oscillators. For networks of chaotic Rössler oscillators formed according to the random link attachment rule, the network collapse phenomenon and its SOC characteristics: (**a**) the critical network size *n*_1_ versus the transient time *T*_*g*_ for different values of the tolerance threshold *δr*_*c*_ and (**b**) distribution of the collapse sizes for Δ*n* ∈ [1, 40]: *p*_*col*_(Δ*n*) ~ Δ*n*^*γ*^ with *γ* ≈ −0.58. Open squares represent the size distribution predicated from the eigenvector analysis. Inset: the linear relation between |*e*_2,*i*_| and *δr*_*i*_ as predicted [Relation (2)]. The data are averaged over 100 network realizations.

We choose the chaotic Rössler oscillator^78^ described by (*dx*/*dt*, *dy*/*dt*, *dz*/*dt*) = (−*y* − *z*, *x* + 0.2*y*, 0.2 + *xz* − 9.0*z*). The oscillators at different nodes are coupled through the *x* variable with the coupling function **H**([*x*, *y*, *z*]^*T*^) = [0, *y*, 0]^*T*^. We define the synchronization error as *δr*_*i*_ = |*x*_*i*_ − 〈*x*〉|. The coupling strength is fixed at *ε* = 0.35. The stable synchronization region from the MSF curve is open at the right side^77^, i.e., the transverse mode *i* is stable for *σ*_*i*_ > *σ*_*l*_ ≈ 0.157. Adopting the random attachment rule, we grow the network under the constraint of synchronization stability and find the phenomenon of network collapse to be robust. For example, Fig. 6(a) shows the critical network size *n*_1_ versus the transient time *T*_*g*_ for different values of the tolerance threshold *δr*_*c*_. We see that, while *n*_1_ increases monotonically with *T*_*g*_ and *δr*_*c*_, the rate is somewhat smaller than that associated with the preferential attachment rule [Fig. 3(b)], indicating that the random attachment rule tends to make network collapses more frequent. Figure 6(b) shows the algebraic distribution of the collapse size generated by parameters *δr*_*c*_ = 1 × 10^−5^ and *T*_*g*_ = 400, which shows *p*_*col*_(Δ*n*) ~ Δ*n*^*γ*^ for Δ*n* ∈ [1, 50], with *γ* ≈ −0.58. These results suggest that the SOC characteristics of the network collapse phenomenon are robust, regardless of the details of the network growth mechanism and of the nodal dynamical processes.

For the randomly growing chaotic Rössler network, we find that the relationship between the synchronization error *δr*_*i*_ and the eigenvector element *e*_2,*i*_ can still be described by (2) [inset in Fig. 6(b)]. However, when analyzing the algebraic size distribution using the eigenvectors, we note that the agreement between the theoretical predication and the direct simulation results is not as good as that for the preferential attachment growth rule. For example, by truncating the eigenvector ***e***_2_ of a random network of *n* = 800 nodes, we obtain 
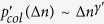
 with *γ*′ ≈ −0.94. The difference in the value of the algebraic scaling exponent can be attributed to the limited size of the network generated subject to the synchronization constraint as well as to the relatively short transient period (small values of *T*_*g*_). In fact, in a computationally feasible implementation of the random growth model with continuous-time dynamics, the largest network generated has the size *n* ≈ 50, rendering somewhat severe the finite size effect. Nonetheless, in spite of the finite-size effect, the SOC features of the network collapse phenomenon are robust.

We note that the transient time for network synchronization, *T*_*s*_, depends on the network size *n* through the eigenvalues of the network coupling matrix. The system size *n*, however, varies with time due to network growth. The transient time *T*_*s*_ also depends on the synchronization threshold *δr*_*c*_ and the initial perturbation *δr*_*o*_. In general, the value of *T*_*s*_ can be estimated numerically. For example, for the coupled logistic map network, during the network growth process the eigenvalue of the most unstable mode (*σ*_2_ or *σ*_*n*_) is on the order of 10^−2^. Our theoretical analysis then gives *T*_*s*_ ≈ [*ln*(*δr*_0_/*δr*_*c*_)]/*σ*_2,*n*_. Since *δr*_0_ ≈ 1 and *δr*_*c*_ ≈ 10^−10^, we have *T*_*s*_ ≈ 300, which is the value used in Figs 1, 2, 3, 4, 5. The transient time for the coupled Rössler oscillator network can be estimated similarly (Fig. 6).

In our study, *T*_*s*_ was introduced mainly for the purpose of distinguishing growing networks from static (*T*_*s*_ = 0) and non-constrained (*T*_*s*_ → ∞) networks. The phenomena observed, i.e., network collapse and self-organized criticality (SOC), are not sensitive to the value of *T*_*s*_. For instance, network growth dynamics similar to that in Fig. 1(a) can be observed for *T*_*s*_ = 50 and 500. It is worth mentioning that, despite progress made in recent years, to predict *precisely* the transient time of synchronization for complex networked dynamical systems remains to be a challenging problem.

## Discussions

Growth, or expansion, is a fundamental feature of complex networks in nature, society, and technological systems. Growth, however, is often subject to constraints. Traditional models of complex networks contain certain growth mechanism, such as one based on the preferential attachment rule^3^, but impose no constraint. Apparently, when growth is constrained, typically the network cannot expand indefinitely, nor can its size be a monotonous function of time. As a result, during the growth process there must be times when the network size is reduced (collapse). But are there generic features of the collapse events? For example, statistically what is the distribution of the collapse size, and are there universal characteristics in the distribution?

This paper addresses these intriguing questions using synchronization as a concrete type of constraint. In particular, taking into account the effects of desynchronization tolerance and synchronization speed, we propose and investigate growing complex networks subject to the constraint of synchronization stability. We find that, as new nodes are continuously added into the network, it can self-organize itself into a critical state where the addition of a single node can trigger a large scale collapse. Statistical analysis of the characteristics of the collapse events such as the degree distribution of the collapsed nodes, the collapse frequency, and the collapse size distribution, indicates that constraint induced network collapse can be viewed as an evolutionary process towards self-organized criticality. The SOC feature is especially pronounced as the collapse size follows an algebraic scaling law. We develop an eigenvector analysis to understand the origin of the network collapse phenomenon and the associated scaling behaviors.

In a modern society, cities and infrastructures continue to expand. In social media, various groups (social networks) keep growing. When constraints are imposed, e.g., manifested as governmental policies or online security rules, how would the underlying network respond? Can constraints lead to large scale, catastrophic collapse of the entire network? These are difficult but highly pertinent questions. Our findings provide some hints about the dynamical features of the network collapse phenomenon, but much further efforts are needed in this direction of complex systems research.

## Methods

### Eigenvector analysis of network synchronizability

Say at step *n*′ of the growth, the network contains *n* − 1 synchronized oscillators and a new oscillator of random initial condition is introduced. Due to the new oscillator, the trajectories of the existing oscillators leave, at least temporarily, the synchronous manifold ***x***_*s*_. Let *δ**x***_*i*_ = ***x***_*i*_ − ***x***_*s*_ be the distance of the *i*th oscillator from the manifold, which is the synchronization error. The evolution of *δ**x***_*i*_ is governed by the following variational equation:


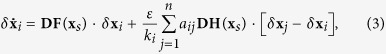


where **DF(*****x***_***s***_) and **DH(*****x***_***s***_) are the Jacobian matrices of the local dynamics and the coupling function evaluated on ***x***_*s*_, respectively. Equation (3) is obtained by linearizing Eq. (1) about the synchronous manifold ***x***_*s*_, which characterizes its local stability^75^. To keep the expanded network synchronizable, a necessary condition is that all the synchronization errors, {*δ**x***_*i*_} approach zero exponentially with time. Projecting *δ**x***_*i*_ into the eigenspace spanned by the eigenvector ***e***_*i*_ of the network coupling matrix *C* = *εa*_*ij*_/*k*_*i*_, we can diagonalize the *n* coupled variational equations into *n* decoupled modes in the blocked form


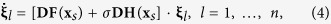


where ***ξ***_*l*_ is the *l*th mode transverse to the synchronous manifold ***x***_*s*_, and 0 = *σ*_1_ > *σ*_2_ … *σ*_*n*_ are the eigenvalues of the coupling matrix *C*. Among the *n* modes, the one associated with *σ* = 0 represents the motion within the synchronous manifold. The network is synchronizable only when all the transverse modes (***ξ***_*j*_, *j* = 2, …, *n*) are stable, i.e., the largest Lyapunov exponent among these modes should be negative: Λ(*σ*) < 0. For typical nonlinear oscillators and smooth coupling functions, previous works^75^,^76^,^77^ showed that Λ(*σ*) can be negative within a bounded region in the parameter space of *σ*, i.e., Λ(*σ*) < 0 for *σ* ∈ (*σ*_*l*_, *σ*_*r*_). Thus, the necessa_*r*_y condition to make the synchronous state stable is *σ*_*l*_ < *σ*_*j*_ < *σ*_*r*_ for all the transverse modes (*j* = 2, …, *n*). For the chaotic logistic map used in our *n*umerical simulations, we have *σ*_*l*_ = 0.5 and *σ*_*r*_ = 1.5.

The eigenvalue analysis, also known as the master stability function (MSF) analysis, is standard in synchronization analysis^75^,^76^. It not only indicates whether a network is synchronizable, but also quantifies the degree of synchronization stability as well as the synchronization speed in certain situations^79^,^80^,^81^. Specifically, by examining the Lyapunov exponents associated with the two extreme modes, Λ(*σ*_2_) and Λ(*σ*_*n*_), one can predict whether the network is synchronizable and how stable (unstable) the synchronous state is. In general, the smaller Λ(*σ*_2_) and Λ(*σ*_*n*_) are, the more stable the synchronous state is^75^,^76^,^77^. Because of the relation Λ(*σ*_2,*n*_) ∝ *σ*_2,*n*_, near the critical points *σ*_*l*_ and *σ*_*r*_, the network synchronizability can be characterized by the stability distances *d*_*l*_ = *σ*_2_ − *σ*_*l*_ and *d*_*r*_ = *σ*_*r*_ − *σ*_*n*_. For a synchronizable network, we have *d*_*l*,*r*_ > 0. Moreover, the larger *d*_*l*_ and *d*_*r*_ are, the more stable the synchronous state will be. Otherwise, if one of the distances is negative, the synchronous state will be unstable. In the asynchronous case, the smaller *d*_*l*_ and *d*_*r*_ are, the more unstable the synchronous state will be.

### Simulations

For the coupled logistic map networks, the dynamics are simulated by iterating the maps in parallel according to Eq. (1). For the coupled Rössler oscillator networks, the 4^th^-order Runge-Kutta method was used to obtain the dynamical evolution of the system with the time step *δt* = 10^−3^.

## Additional Information

**How to cite this article**: Wang, Y. *et al*. Growth, collapse, and self-organized criticality in complex networks. *Sci. Rep*. **6**, 24445; doi: 10.1038/srep24445 (2016).
